# Alteration of synaptic connectivity of oligodendrocyte precursor cells following demyelination

**DOI:** 10.3389/fncel.2015.00077

**Published:** 2015-03-17

**Authors:** Aurélia Sahel, Fernando C. Ortiz, Christophe Kerninon, Paloma P. Maldonado, María Cecilia Angulo, Brahim Nait-Oumesmar

**Affiliations:** ^1^INSERM U1127, Institut du Cerveau et de la Moelle EpinièreParis, France; ^2^Université Paris 6, Sorbonne Paris Cité, UMR-S1127Paris, France; ^3^Centre National de la Recherche Scientifique UMR 7225Paris, France; ^4^INSERM U1128Paris, France; ^5^Université Paris Descartes, Sorbonne Paris CitéParis, France

**Keywords:** NG2 cells, oligodendrocyte precursor cells, oligodendrocyte, neuron-OPC synapses, demyelination, multiple sclerosis

## Abstract

Oligodendrocyte precursor cells (OPCs) are a major source of remyelinating oligodendrocytes in demyelinating diseases such as Multiple Sclerosis (MS). While OPCs are innervated by unmyelinated axons in the normal brain, the fate of such synaptic contacts after demyelination is still unclear. By combining electrophysiology and immunostainings in different transgenic mice expressing fluorescent reporters, we studied the synaptic innervation of OPCs in the model of lysolecithin (LPC)-induced demyelination of *corpus callosum*. Synaptic innervation of reactivated OPCs in the lesion was revealed by the presence of AMPA receptor-mediated synaptic currents, VGluT1+ axon-OPC contacts in 3D confocal reconstructions and synaptic junctions observed by electron microscopy. Moreover, 3D confocal reconstructions of VGluT1 and NG2 immunolabeling showed the existence of glutamatergic axon-OPC contacts in post-mortem MS lesions. Interestingly, patch-clamp recordings in LPC-induced lesions demonstrated a drastic decrease in spontaneous synaptic activity of OPCs early after demyelination that was not caused by an impaired conduction of compound action potentials. A reduction in synaptic connectivity was confirmed by the lack of VGluT1+ axon-OPC contacts in virtually all rapidly proliferating OPCs stained with EdU (50-ethynyl-20-deoxyuridine). At the end of the massive proliferation phase in lesions, the proportion of innervated OPCs rapidly recovers, although the frequency of spontaneous synaptic currents did not reach control levels. In conclusion, our results demonstrate that newly-generated OPCs do not receive synaptic inputs during their active proliferation after demyelination, but gain synapses during the remyelination process. Hence, glutamatergic synaptic inputs may contribute to inhibit OPC proliferation and might have a physiopathological relevance in demyelinating disorders.

## Introduction

Demyelination is defined by the loss of the myelin sheath insulating nerve fibers. The important consequences of demyelination are imposed on the axon in the form of disturbed conduction and compromised survival. In a process referred to as remyelination, the central nervous system (CNS) has the capacity to restore myelin sheaths to demyelinated axons enabling them to recover conduction of action potentials and to provide effective neuroprotection (Franklin and Ffrench-Constant, 2008). This regenerative process is mainly mediated by the endogenous oligodendrocyte precursor cells (OPCs) expressing the proteoglycan NG2 (Nishiyama et al., 2009), that serve as a major source of remyelinating oligodendrocytes in demyelinating diseases, such as multiple sclerosis (MS). However, in MS, the extent of remyelination often fails, leading to chronically demyelinated lesions with substantial axonal loss (Trapp et al., 1998). The reasons why remyelination fails in MS are not completely understood, although the complex pathological environment within the lesion is probably a major cause. Understanding the mechanisms that control proliferation and differentiation of OPCs in demyelinating conditions is an exciting challenge as it may lead to enhanced myelin repair. This is also of significant clinical interest as it might open up perspectives for new remyelinating therapies.

Cells of the oligodendroglia lineage express different types of functional glutamatergic receptors in the living tissue such as AMPA, NMDA, kainate and metabotropic glutamate receptors (Berger et al., 1992; Matute, 1998; Yuan et al., 1998; Ziak et al., 1998; Karadottir et al., 2005; Salter and Fern, 2005; Kukley and Dietrich, 2009; Haberlandt et al., 2011). However, only scant information exists on the activation modes of most receptors. While AMPA receptors in OPCs are known to be activated through synaptic release from neurons (Bergles et al., 2000), other receptors are likely activated by extrasynaptic mechanisms that still need to be identified (Maldonado and Angulo, 2014). Interestingly, OPCs in both gray and white matter receive functional AMPA receptor-mediated synapses from neurons (Bergles et al., 2000; Kukley et al., 2007; Ziskin et al., 2007). Most studies have shown that OPCs receive synaptic inputs from unmyelinated axons and express Na^+^ conductances, though they are unable to trigger action potential firing (De Biase et al., 2010; Kukley et al., 2010; Maldonado et al., 2011; Sun and Dietrich, 2013). Nevertheless, OPCs do not necessarily have the same intrinsic electrophysiological properties in young and adult mice, supporting the idea that the properties of an existing population of OPCs change during development and give raise to distinct adult OPCs (Maldonado et al., 2013). Whether modifications of intrinsic properties arise in adult white matter OPCs in pathological conditions is currently unknown. The role of neuron-OPC synapses also remains unclear.

In the *corpus callosum*, unmyelinated axons establish glutamatergic synapses with OPCs as early as the first postnatal week and synaptic connectivity increases in the adult (Kukley et al., 2007; Ziskin et al., 2007; De Biase et al., 2010). After a demyelinating injury, demyelinated axons also form functional synapses with a minor pool of OPCs derived from the subventricular zone (SVZ) that contribute to oligodendrocyte regeneration (Etxeberria et al., 2010). However, whether all endogenous OPCs within demyelinated lesions are contacted by synapses and whether these synapses are regulated after demyelination is still unknown. In the present study, we use a model of lysolecithin (LPC)-induced focal demyelination of the *corpus callosum* in mice expressing specific fluorescent reporters to analyse glutamatergic innervation of reactivated OPCs, which are characterized by higher proliferation and migration properties following injury. Virtually all recorded OPCs in control and LPC-induced lesions display Na^+^ currents and no changes in voltage-independent K^+^ conductances. Reactivated OPCs exhibit synaptic currents sensitive to the AMPA receptor antagonist NBQX. They also were characterized by the presence of VGluT1+ puncta in mouse LPC-induced demyelinating lesions and in MS tissue. Importantly, a drastic down-regulation of functional glutamatergic synapses occurs during the active proliferation following demyelination in the mouse LPC-induced lesions.

## Materials and methods

### LPC-induced demyelination

**Figure 1:**
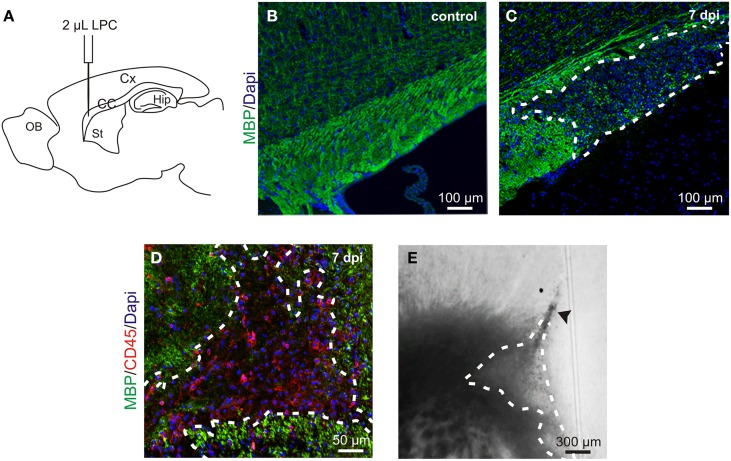
**LPC-induced demyelination model. (A)** Lysolecithin (LPC) was injected in the *corpus callosum* (CC) under anesthesia in a stereotaxic apparatus (coordinates respect to bregma: 1 mm lateral, 1.5 mm rostral; 1.8 mm depth to brain surface); Cx, cortex; Hip, hippocampus; St, striatum; OB, olfactory bulb. **(B,C)** Sagittal slices of a healthy control *corpus callosum* **(B)** and an LPC-induced lesion at 7 days post injection (dpi) **(C)** stained with MBP (green) and Dapi (blue). **(D)** Sagittal slice of an LPC-induced lesion in the mouse *corpus callosum* at 7 dpi stained with MBP (green), CD45 (red) and DAPI (blue). **(E)** DIC video microscopy of the LPC lesion (dashed lines) at 7 dpi in a coronal acute *corpus callosum* slice. Note the trace of the injection pipette (arrowhead).

All experiments followed European Union and institutional guidelines for the care and use of laboratory animals. Histochemical and electrophysiological experiments were performed with transgenic mice used at adult heterozygous stages: NG2-DsRed (Ziskin et al., 2007), PDGFRα-GFP (Hamilton et al., 2003), CNPase-GFP (Yuan et al., 2002) and Cx3CR1-GFP (Jung et al., 2000). Wild-type (Wt) C57BL/6 adult mice were also used for histological analysis of VGluT1 on NG2+ cells. Focal demyelinating lesions were induced by a stereotaxic injection of 2 μl lysolecithin solution (LPC, Sigma, 1% LPC in 0.9% NaCl) in the *corpus callosum* in single or double adult (PN40-PN70) transgenic mice anesthetized with Ketamine (0.1 mg/g) and Xylazine (0.01 mg/g) as previously described (coordinates: 1 mm lateral, 1.5 mm rostral to Bregma, and 1.8 mm depth to brain surface; Figure 1A, (see also Tepavcevic et al., 2011). Control mice were injected with saline solution only.

### Acute slice preparation and electrophysiology

Acute coronal slices (300 μm) of LPC-injected *corpus callosum* were prepared from different mouse strains following previously described procedures (Vélez-Fort et al., 2010). Briefly, patch-clamp recordings were performed at 33°C using an extracellular solution containing (in mM): 126 NaCl, 2.5 KCl, 1.25 NaH2PO4, 26 NaHCO3, 20 glucose, 5 pyruvate, 2 CaCl2 and 1 MgCl2 (95% O2, 5% CO2). The intracellular solution contained (in mM): 130 Cs-gluconate, 10 4-aminopyridine, 5 tetraethylammonium chloride, 5 EGTA, 0.5 CaCl2, 2 MgCl2, 10 HEPES, 2 Na2-ATP, 0.2 Na-GTP, and 10 Na2-phosphocreatine (pH ≈ 7.4, 296 mOsm). Potentials were corrected for a junction potential of −10 mV. Whole-cell recordings of OPCs were obtained using Multiclamp 700B, filtered at 4 kHz and digitized at 20 kHz. Digitized data were analyzed off-line using pClamp10.1 (Molecular Devices) and Spacan, a collection of IGOR Pro functions (Dugue et al., 2009). In our conditions, the input resistance of OPCs was 5.03 ± 0.57 GΩ (*n* = 23), 5.21 ± 0.93 GΩ (*n* = 16), 5.25 ± 0.81 GΩ (*n* = 42) and 4.94 ± 0.76 GΩ (*n* = 46) in control, at 4, 7, and 14 days post injection (dpi), respectively (*p* > 0.05). The voltage-independent K^+^ current density for each cell was calculated by dividing the K^+^ current amplitude obtained at −120 mV by its capacitance. The Na^+^ current density for each cell was calculated by dividing the Na^+^ current amplitude obtained after leak subtraction at −10 mV by its capacitance. It is noteworthy that pericytes express DsRed in NG2-DsRed mice (Supplementary Figure 1A). These cells are easily discarded for recordings by their bipolar shape that line blood vessels visible in DIC, as we previously performed (Vélez-Fort et al., 2010; Maldonado et al., 2013). Spontaneous and miniature synaptic currents of OPCs were recorded at a holding potential of −90 mV and detected with a threshold of 3 times the noise standard deviation during a time window of 2 min for controls, 7 and 14 dpi and of 5 min for 4 dpi. We verified that there is no correlation between the frequency of the spontaneous currents and the noise standard deviation either when plotting the data of all recorded cells together or when plotting the data separately for each data group (*p* > 0.05). In addition, no differences of the mean of noise standard deviation were observed among groups (2.4 ± 0.1 pA, 2.3 ± 0.1 pA, 2.5 ± 0.3 pA, 2.3 ± 0.1 pA for control, 4, 7, and 14 dpi, respectively; *p* > 0.05). The lack of synaptic currents in cells without synaptic activity was confirmed by bath application of the potent secretagogue ruthenium red (75 μM). Compound action potentials (CAPs) were obtained by stimulating white matter fibers at two different positions with a monopolar tungsten electrode while a recording electrode (glass pipette) was placed in the lesion core (100 μs stimulations; Iso-Stim 01D, npi electronic GmbH, Tamm, Germany). This allowed us to calculate the conduction velocity (Vc) as follows: Vc = D1-D2/L, where D1 and D2 correspond to the longer and shorter distance between the stimulation and recording electrode, respectively, and L to the latency between CAP onsets obtained for the two electrode positions.

### Immunohistochemistry, EDU treatment and electron microscopy

Adult mice were anesthetized and transcardially perfused with 2% paraformaldehyde. Brains were dissected and post fixed for 2–4 h, cryoprotected in 20% sucrose and stained using standard protocols. The following primary antibodies were used: rabbit anti-NG2 (1:200; Millipore), rabbit anti-Olig2 (1:200; Millipore), mouse anti-Olig1 (1/100, R and D Systems), mouse anti-CC1 (1:100; Abcam), mouse anti-VGluT1 (1:500; Millipore), rabbit anti-Iba1 (1:100; Millipore), rat anti-PDGFRα (1:200; Santa Cruz) and rabbit anti-MBP (1:100; Millipore). Edu (Invitrogen), a BrdU analog, was injected intraperitoneally (75 mg/kg) every 2 h for 10 h before sacrifice at 4 dpi. Edu staining was detected with the Click-It™ Kit (Invitrogen). For electron microscopy analysis, Wt brains were processed as previously described (Tepavcevic et al., 2011) and imaged using a Siemens electron microscope.

### Confocal microscopy analysis

Images were acquired using an Olympus confocal microscope or a Zeiss apotome system (AxoVision LE Rel 4.5) and processed using Axovision, ImageJ, Adobe Photoshop/Illustrator (Adobe Systems) and Volocity (3D images; PerkinElmer). For quantitative analysis, the number of reporter+ oligodendroglial cells expressing NG2, CC1, or Olig2, was counted and expressed as a percentage of the total number of reporter+ cells. To visualize the co-localization between VGluT1 puncta and NG2 labeling in lesions, we used 3D reconstructions (z-stack of 6 μm; 0.2 μm z-step). Single OPCs were isolated and VGluT1 puncta were filtered according to a size ranging from 0.3–0.7 μm in diameter (Herzog et al., 2011).

### MS tissue samples

Autopsy brain samples from eight MS patients with confirmed secondary progressive (SP, *n* = 7) and relapsing progressive (RP, *n* = 1) disease course, and three control cases without neurological diseases, were obtained from the British MS Tissue Bank (collaboration with Dr R. Reynolds, London) and the French Brain Tissue Bank GIE-NeuroCEB (Hôpital Pitié-Salpêtrière, France). All MS lesions were characterized using Luxol fast blue/MHCII (macrophages/microglia) staining and classified according to their inflammatory activity and on the basis of histological criteria of acute lesions (active demyelination, myelin vacuolation, inflammation or edema, minor gliosis and vague margin), chronic lesions (no myelin vacuolation, absence of inflammation, gliosis, axonal loss and sharp margin) and shadow plaques (myelin pallor; Lassmann, 1998). Immunohistochemistry of NG2 and VGluT1 and 3D reconstructions of cells were performed as described above. VGluT1+ punctas per OPC were analyzed in active, chronic active and shadow plaques, as well as in the normal appearing white matter of controls and MS cases.

### Statistical analysis

All values are expressed as mean ± SEM. Each data group was first subject to D'Agostino and Pearson normality Test. According to the data structure, multiple group comparisons were done using either One Way ANOVA or Kruskal-Wallis test. Bonferroni or Dunn's multiple comparison *post-hoc* tests were used respectively. All statistical tests were performed with GraphPad Prism 5.00 software (GraphPad Software Inc., USA). Correlation was tested with the Spearman *r*-test.

## Results

### Transgenic mouse strategy to identify OPCs in acute slices of the demyelinated mouse *corpus callosum*

Demyelinating lesions, induced by stereotaxic injection of LPC into the adult mouse *corpus callosum* (Figure 1A), were defined by the loss of myelin (Figures 1B,C) and by an increased number of inflammatory cells (Figure 1D). To analyze the electrophysiological properties of OPCs following demyelination, we performed coronal acute brain slices in control and after demyelination. The demyelinated area in *corpus callosum* was identified with DIC videomicroscopy at low magnification as a brighter region than the normal white matter surrounding it (Figure 1E).

**Figure 2:**
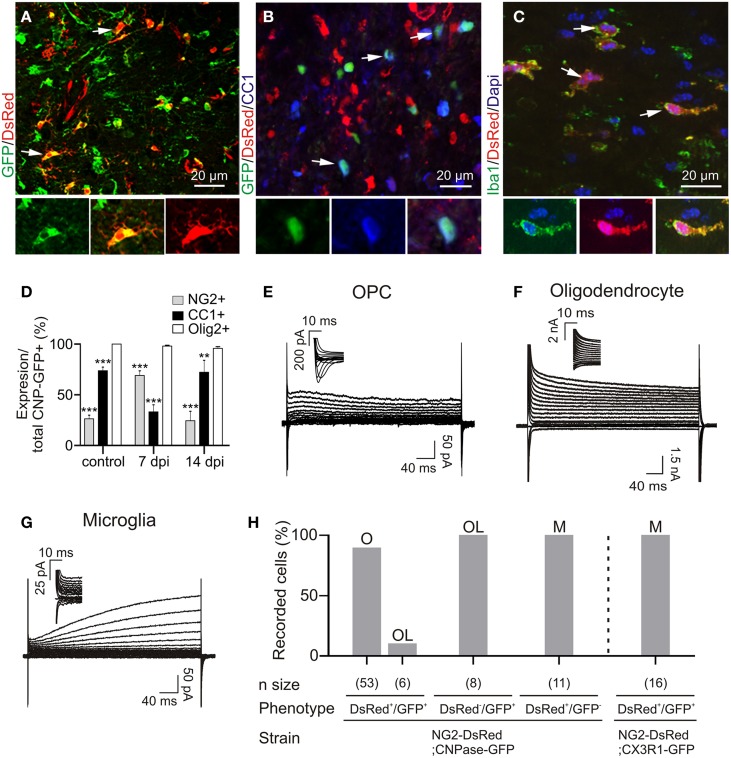
**Immuno-characterization and electrophysiological properties of OPCs, oligodendrocytes and microglia in transgenic lines following demyelination. (A)** OPCs were identified as DsRed+/GFP+ cells (arrows) in the NG2-DsRed;CNPase-GFP double transgenic mouse. **(B)** Mature oligodendrocytes were identified as CC1+ cells (blue) and also expressed GFP (arrows) but not DsRed. **(C)** Iba1+ resident microglia/macrophage expressing DsRed in the NG2-DsRed mouse strain. **(D)** Bar plot showing the percentage of NG2+ and CC1+ cells within control and lesioned *corpus callosum* in CNPase-GFP animals (*N* = 3 mice). **(E)** Currents elicited by voltage steps from +40 mV to −120 mV in a DsRed+/GFP+ OPC. held at −90 mV and recorded inside a lesion. Note the presence of INa^+^ (inset). **(F)** Currents induced by voltage steps from +40 mV to −120 mV in a DsRed−/GFP+ oligodendrocyte at 14 dpi held at −90 mV. **(G)** Currents induced by voltage steps from +40 mV to −120 mV in a DsRed+/GFP− microglia held at −90 mV. Note the absence of INa^+^ (inset). We confirmed that DsRed was expressed in activated microglia inside lesions by crossing NG2-DsRed line with the CX3CR1-EGFP strain in which microglia/macrophages express GFP (see Figure 1H) (Avignone et al., 2008). **(H)** Bar plot for the proportion of OPCs (O), microglia (M) and oligodendrocytes (OL) identified by their electrophysiological profiles and recorded in different mouse strains following demyelination. Scale bars for insets: 10 μm. ^**^p < 0.01, ^***^p < 0.001 respect to Olig2 expression.

All patch-clamp recordings were performed inside the lesion core in adult NG2-DsRed;CNPase mice. This double transgenic line allowed us to unambiguously discriminate OPCs and oligodendrocytes in brain slices and compared their electrophysiological properties in lesions, as all cells of the oligodendroglial lineage express GFP in the CNPase-GFP mouse line (Yuan et al., 2002). The large majority of DsRed+/GFP+ cells in the lesion had the immunohistochemical phenotype of NG2+ OPCs (Figure 2A) while DsRed−/GFP+ cells were labeled for CC1, a specific marker of differentiated oligodendrocytes (Figure 2B). As expected in the LPC model of demyelination (Watanabe et al., 2002), the population of DsRed+/GFP+ OPCs was largely increased at 7 dpi whereas a clear increase in differentiated DsRed−/GFP+/CC1+ oligodendrocytes was detected at 14 dpi (Figure 2D). In addition to DsRed+/GFP+ OPCs and DsRed−/GFP+ oligodendrocytes, we observed the presence of DsRed+/GFP- cells with large somata and thick primary processes inside the lesion, characteristic of activated microglia. To confirm the expression of DsRed by microglia in LPC-induced lesions, we used the microglial marker Iba1 and confirmed the expression of DsRed in Iba1+ cells (Figure 2C, see also Bu et al., 2001). Hence, in LPC-induced lesions, the NG2-DsRed;CNPase-GFP mouse line allows for the unequivocal identification of OPCs from mature oligodendrocytes and microglia by the simultaneous expression of both DsRed and GFP.

Reporter+ cells were recorded with a Cs-gluconate-based intracellular solution at −90 mV in acute brain slices. We found that 90% of DsRed+/GFP+ cells show a voltage-dependent current profile characteristic of OPCs with outwardly rectifying steady-state currents and inward Na^+^ currents (Figures 2E,H). The remaining 10% of DsRed+/GFP+ cells and all DsRed−/GFP+ cells had the linear current profile of mature oligodendrocytes (Kukley et al., 2010) with large K^+^ currents insensitive to intracellular Cs^+^ (Figures 2F,H). Finally, DsRed+/GFP- cells, excluding pericytes (see Materials and Methods), expressed a time and voltage-dependent outward current and lacked inward Na^+^ currents (Figures 2G,H), typical of activated microglia in cell culture (Klee et al., 1999). To further confirm the expression of DsRed by microglia in LPC-induced lesions, we also performed patch-clamp recordings of DsRed+/GFP+ cells in LPC lesions of the double transgenic NG2-DsRed;Cx3CR1-GFP (microglia marker) mice, and confirmed that all double reporter+ cells had the above described microglia electrophysiological phenotype (Figure 2H). Hence, the electrophysiological characteristics of different reporter+ cells inside the lesion perfectly match the immunohistochemistry analysis. The major characteristic distinguishing OPCs from microglia and oligodendrocytes in lesions is the presence of Na^+^ currents, similarly to previous observations on OPCs in normal conditions (De Biase et al., 2010; Kukley et al., 2010).

In the healthy adult brain of this double transgenic line, DsRed was predominantly expressed in pericytes (Supplementary Figure 1A). Very few DsRed+/GFP+ OPCs were detected in both white and gray matter regions, particularly after PN45, and the large majority of DsRed−/GFP+ cells were mature oligodendrocytes (Figures 2B,F). Therefore, to recognize OPCs in healthy white matter, we used the PDGFRα-GFP mouse line. Immunohistochemical analysis showed that 50% of GFP+ cells expressed NG2 (Supplementary Figures 1B,D), while the remaining cells were mature CC1+ oligodendrocytes (Supplementary Figures 1C,D; Clarke et al., 2012). Consistent with this data, patch-clamp recordings from the non-demyelinated *corpus callosum* of PDGFRα-GFP mice revealed that 52% of recorded GFP+ cells had the typical current profile of OPCs with Na^+^ currents (Supplementary Figures 1E,G); the remaining 48% showing the classical linear phenotype of mature oligodendrocytes (Supplementary Figures 1F,G; see also Kukley et al., 2010). Therefore, our strategy was to use PDGFRα-GFP line as controls and NG2-DsRed;CNPase-GFP line to record reactivated OPCs, mature oligodendrocytes and activated microglia after LPC-induced demyelination.

**Figure 3:**
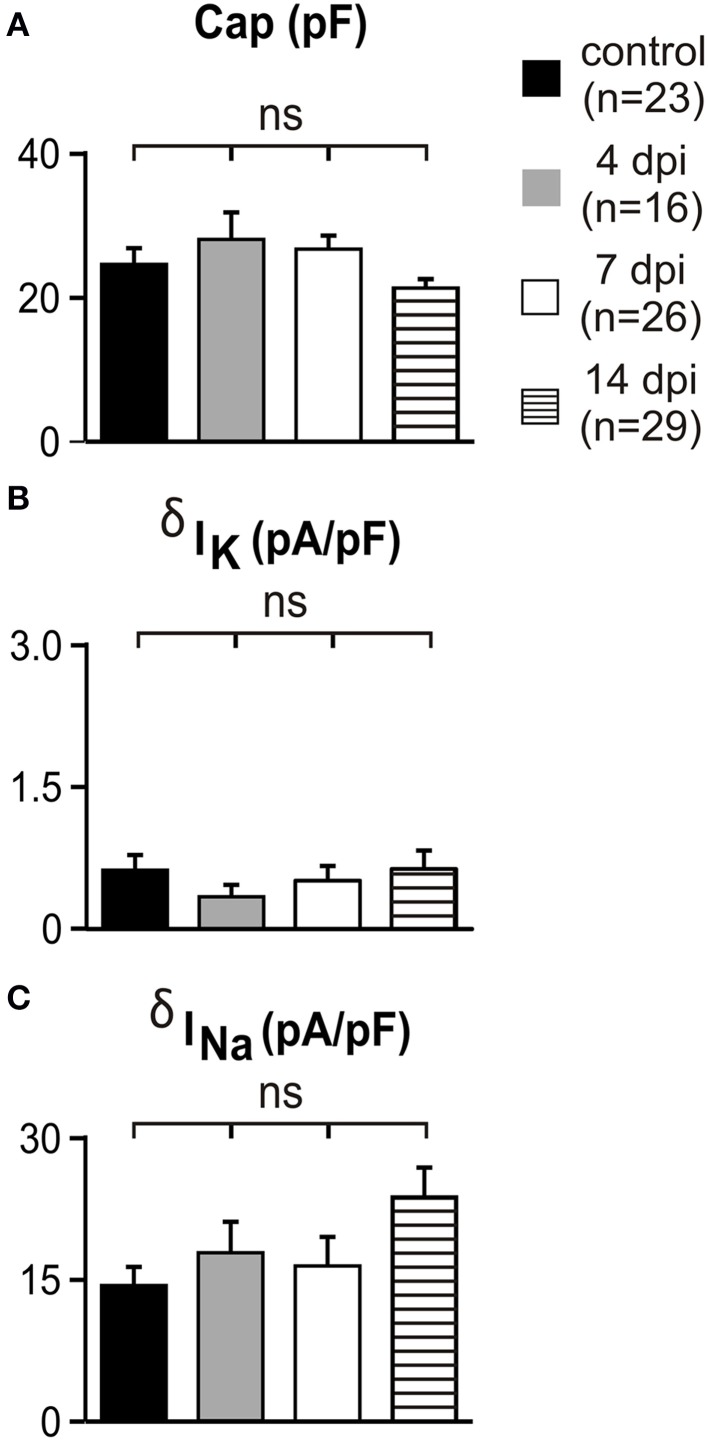
**Voltage-independent K^+^ currents and Na^+^ currents did not change in white matter OPCs following demyelination**. (A–C) Capacitance, K^+^ and Na^+^ current densities of OPCs recorded in control conditions and in lesions at different dpi. K^+^ and Na^+^ current densities were calculated at −120 mV and −10 mV, respectively (see Materials and Methods). Note that the scale is 10 times smaller for K^+^ than for Na^+^ current densities. ns, not significant.

In order to reveal any potential upregulation of voltage-independent K^+^ conductances in OPCs after demyelination as reported during gray matter development (Maldonado et al., 2013) and to test for potential modifications on Na^+^ channel-mediated current amplitudes in reactived OPCs, we compared the capacitance, K^+^ and Na^+^ current densities between controls and at 4, 7, and 14 dpi (Figures 3A–C). No differences were detected in lesions with respect to controls and at any time point after LPC injection. Overall, our results indicate that appropriate transgenic lines are needed to ensure OPC, oligodendrocyte and microglia identification under pathological conditions and that Na^+^ and voltage-independent K^+^ conductances of OPCs are not affected by LPC-induced lesions.

### OPCs receive glutamatergic synaptic inputs in demyelinated lesions

**Figure 4:**
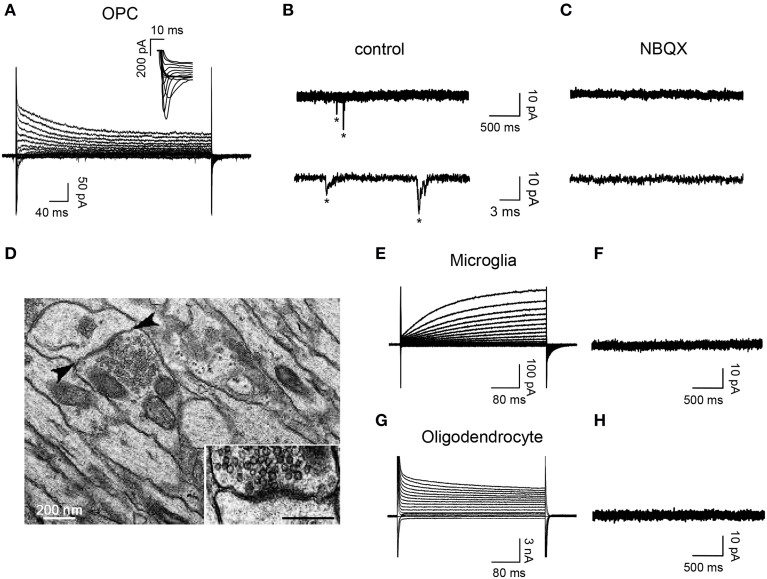
**OPCs display NBQX-sensitive spontaneous synaptic activity while microglia and oligodendrocytes completely lack synaptic events. (A)** Currents elicited in an OPC at 7 dpi by voltage steps from +40 to −120 mV. Note the presence of INa^+^ (inset). **(B,C)** Spontaneous synaptic currents (^*^) recorded at −90 mV from the same OPC in control (**B**, frequency: 1.34 Hz) and after bath applying 10 μM NBQX (**C**, frequency: 0 Hz). **(D)** Electron-micrograph of a LPC lesion at 14 dpi, illustrating a typical synaptic contact between an axon and a putative OPC process (arrowheads, inset). Note that the postsynaptic process lacks gliofilaments and lipofuscin granules, two typical features of astrocytes and microglia, respectively. **(E,G)** Currents induced by voltage steps from +40 to −120 mV in a microglia at 7 dpi **(E)** and an oligodendrocyte at 14 dpi **(G)** held at −90 mV. **(F,H)** Currents recorded at −90 mV from the same cells. Note the lack of synaptic events in both cells types (*n* = 12 and *n* = 14 for microglia and oligodendrocytes, respectively). Scale bar for inset: 200 nm.

To determine the presence of synaptic connectivity in reporter+ cells of demyelinated lesions, we recorded DsRed+/GFP+ OPCs, DsRed+/GFP− microglia and DsRed−/GFP+ oligodendrocytes in slices from 7 dpi NG2-DsRed;CNPase-GFP mice (Figure 4). As expected from previous studies (Kukley et al., 2007; Ziskin et al., 2007; Etxeberria et al., 2010), spontaneous synaptic activity blocked by the AMPA/kainate receptor antagonist NBQX was recorded only in reactivated OPCs (Figures 4A–C). Neither activated microglia, (*n* = 12; Figures 4E,F) nor mature oligodendrocytes (*n* = 14; Figures 4G,H) exhibited spontaneous synaptic currents in lesions, even in the presence of 75 μM ruthenium red (*n* = 4 for each cell type). Therefore, our electrophysiological data demonstrate that OPCs display NBQX-sensitive spontaneous synaptic activity while microglia and mature oligodendrocytes completely lack synaptic events. The existence of synaptic junctions in *corpus callosum* lesions was corroborated by electron microscopy analysis. Figure 4D illustrates the ultra-structural anatomy in a lesion of a synaptic contact between an axon and a putative OPC process distinguished by the lack of gliofilaments and lipofuscin granules, two typical features of astrocytes and microglia respectively. This synapse is characterized by a rigid parallel apposition of membranes, an accumulation of small and round pre-synaptic vesicles and an electron-dense post-synaptic active zone (Figure 4D, inset), which are typical features of asymmetric excitatory synapses (Kukley et al., 2007; Ziskin et al., 2007; Harris and Weinberg, 2012).

**Figure 5:**
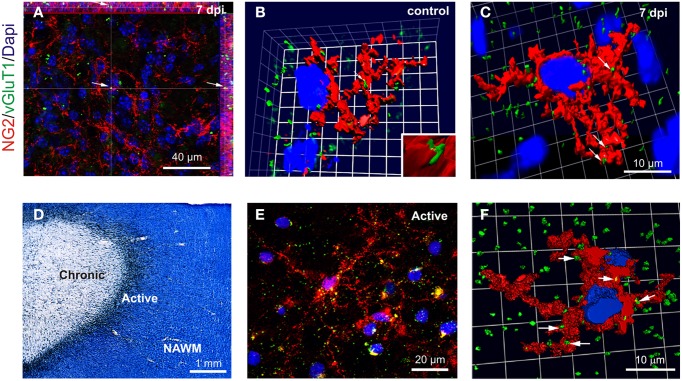
**VGluT1+ a xon-OPC contacts in LPC-demyelinated and MS lesions. (A)** Immunohistochemistry showing NG2 (red) and VGluT1 (green) labeling in a LPC-induced lesion at 7 dpi. Arrow indicates a VGluT1 puncta. **(B,C)** 3D reconstruction of NG2 (red) and VGluT1 (green) labeling in control **(B)** and LPC-induced lesions at 7 dpi **(C)**. Contacts are indicated by arrows. **(D)** MS brain section stained with Luxol fast blue/MHCII (black), illustrating a typical chronic active lesion in the subcortical white matter. The different areas of the lesion were classified as active, chronic silent and normal appearing white matter (NAWM). Active borders of chronic active lesions were filled with MHCII+ macrophages/microglia, while chronic silent cores were devoid of labeling. **(E)** Immunohistochemistry showing NG2 (red) and VGluT1 (green) staining in the active border of a chronic active lesion. **(F)** 3D reconstruction of a NG2 cell (red) with VGluT1 puncta (green, arrows) in the active border of a lesion. Nuclei were stained with Dapi (blue).

To complement our electrophysiological data on OPC synaptic connectivity, we visualized glutamatergic contacts of OPCs, using 3D confocal reconstructions of VGluT1 and NG2 immunolabeling in control *corpus callosum* and within demyelinated lesions at 7 dpi (Figures 5A–C). NG2+ OPCs were distinguished from activated microglia/macrophages based on their typical amoeboid morphology (data not shown). Our data revealed numerous VGluT1+ puncta on OPC in both the control tissue and within the lesion at 7 dpi (Figures 5A–C). We also examined VGluT1 and NG2 expression in MS post-mortem brain samples. MS lesions were first classed as active, chronic active, chronic silent, shadow plaques and normal appearing white matter (NAWM) according to Luxol-fast blue/MHCII staining. Figure 5D illustrates a typical chronic active lesion in the subcortical white matter with a typical silent core and an active border filled with MHCII+ microglia/macrophages. Interestingly, 3D reconstruction of VGluT1 and NG2 immunostaining in MS brain sections (Figures 5E,F) revealed also glutamatergic VGluT1+ puncta on NG2+ OPCs in active lesions as well as in active borders of chronic lesions (Figure 5F). It is noteworthy that NG2+ cells in these human brain samples also expressed the specific oligodendroglial marker Olig1, which never co-localized with Iba1 (Supplementary Figure 2). Therefore, our data demonstrate the presence of glutamatergic axon-OPC contacts both in mouse LPC-induced demyelinated lesions and in active MS lesions.

### Regulation of axon-OPC synaptic activity in LPC-induced demyelinating lesions

**Figure 6:**
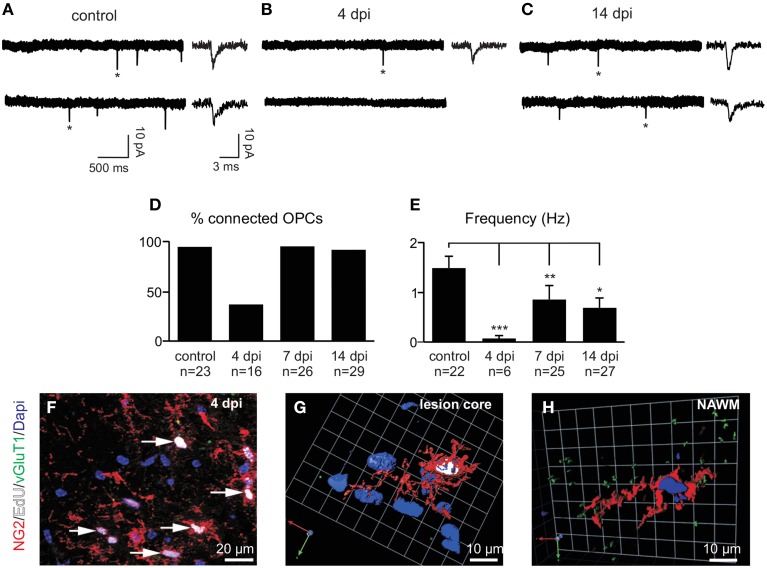
**Spontaneous glutamatergic synaptic currents of OPCs in demyelinated lesions. (A–C)** Spontaneous synaptic currents of recorded OPCs held at −90 mV from a control **(A)**, at 4 **(B)** and 14 dpi **(C)**. Note the fast rise and decay times of individual currents (^*^) in all conditions (insets, see also Figures 7A,C). **(D)** Histogram of the percentage of synaptically connected OPCs in control and at 4, 7, and 14 dpi. **(E)** Bar plot of the frequency of spontaneous synaptic currents observed in connected OPCs in control and at 4, 7, and 14 dpi. Cells without synaptic currents were excluded. ^*^p < 0.05, ^**^p < 0.01, ^***^p < 0.001 respect to the control **(F)** Triple immunolabeling for NG2 (red, arrows), EdU (white) and VGluT1 (green) in a LPC-induced lesion at 4 dpi. **(G)** 3D reconstruction of a typical NG2+ (red), EdU+ (white) cell lacking VGluT1 contacts (green) within the lesion at 4 dpi. **(H)** 3D reconstruction of an NG2+ EdU- cell in the non-demyelinated area of *the corpus callosum* (normal appearing white matter, NAWM). Nuclei were stained with Dapi.

To test whether synaptic properties of reactivated OPCs are modified in LPC lesions, we recorded spontaneous currents of OPCs in slices from control animals and at 4, 7, and 14 dpi which correspond to major phases of OPC proliferation and differentiation inside the lesion (Nait-Oumesmar et al., 1999). At 4 dpi, most cells were weakly or not connected, suggesting that newly generated OPCs may receive few or no synaptic contacts inside the lesion (Figures 6A,B). The proportion of innervated OPCs in control brain slices was 97% (Figure 6D). Interestingly, this proportion fell to 38% at 4 dpi (Figure 6D) and the frequency of spontaneous synaptic events recorded in innervated OPCs at this stage was also greatly reduced (Figures 6B,E). It is also noteworthy that the reduction in synaptic activity at 4 dpi might be caused by a decrease of membrane time and space constants and therefore by a filtering of postsynaptic currents in recorded cells. However, in our recording conditions, we did not observe any change in capacitance, membrane time constant or input resistance with respect to controls and no correlation was obtained between synaptic current frequency and input resistance (Spearman r: −0.116; *p* > 0.05). In addition, bath application of 75 μM ruthenium red alone did not reveal any synaptic current in OPCs lacking synaptic activity at 4 dpi (*n* = 5). Hence, to determine whether this loss of synaptic activity of OPCs at 4 dpi is correlated with active proliferation of endogenous OPCs, lesioned mice were injected with EdU (5 injections of EdU at 2 h intervals before sacrifice) in order to label actively proliferating cells. Our results show that all EdU+/NG2+ OPCs within the lesion virtually lacked VGluT1+ contacts (Figures 6F,G). In contrast, NG2+ OPCs with VGluT1 puncta detected in the normal appearing white matter were not labeled with EdU (Figure 6H). Altogether, these results show a strong down-regulation of synaptic inputs in actively proliferating OPCs in demyelinated lesions.

**Figure 7:**
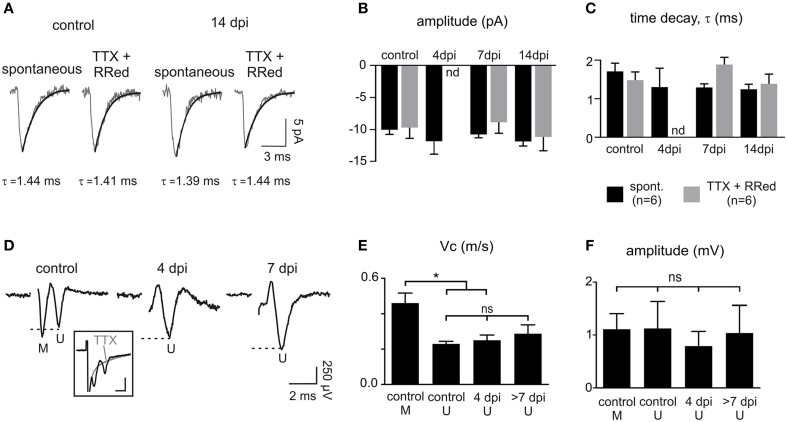
**Properties of OPC synaptic currents and CAPs following demyelination. (A)** Averaged time course of spontaneous and miniature synaptic events in OPCs held at −90 mV in a control and at 14 dpi. Miniature synaptic currents were recorded in the presence of 1 μM TTX and 75 μM ruthenium red as previously described (Vélez-Fort et al., 2010). **(B,C)** Comparison of the amplitude **(B)** and decay time **(C)** of spontaneous and miniature synaptic currents. The large decrease of connected OPCs and the low frequency of connected cells at 4 dpi (see Figures 6D,E) precluded the detection of miniature events at this time point (nd: not determined). **(D)** CAPs in control and in lesions at 4 and 7 dpi obtained after subtracting averaged traces before and after application of 1 μM TTX (3–5 sweeps; inset). Note that the first peak corresponding to myelinated fibers (M) is lost following demyelination. Inset scale bar: 1 mV; 500 μs. **(E,F)** Bar plots of the amplitude **(E)** and conduction velocity **(F)** of myelinated (M) and unmyelinated (U) components of CAPs in control, 4 dpi and after 7 dpi. ^*^p < 0.05, respect to the control. ns, not significant.

After 4 dpi, the proportion of connected OPCs recovered to more than 90% at 7 dpi and remained high at 14 dpi (Figures 6C,D). Even though the proportion of connected OPCs recovered, the frequency of synaptic currents did not increase back to control levels (Figures 6D,E). Nevertheless, the averaged amplitude and kinetics of spontaneous synaptic events were similar at all-time points with respect to controls and equally similar to those of miniature synaptic events, recorded in the presence of TTX and ruthenium red (Figures 7A–C). This implies that spontaneous synaptic events correspond to currents generated by release of single vesicles. Estimated quantal size was around 10 pA at a holding potential of −90 mV in both the control and lesioned brains. Differences in both the proportion of connected cells at 4 dpi and the frequency of synaptic events at 4, 7, and 14 dpi are thus not explained by a decrease in the quantal size after demyelination.

Finally, we asked whether this deficit might result from a lack of axonal conduction in the lesion after demyelination by analyzing extracellular compound action potentials (CAPs). Callosal stimulation in control tissue elicited field responses characterized by two waves with different conduction velocities corresponding to myelinated and unmyelinated fibers (conduction velocities of 0.46 ± 0.05 m.s^−1^ and 0.23 ± 0.01 m.s^−1^, respectively (*n* = 5); Figures 7D,E; see Materials and Methods). As expected for a demyelinating lesion, only the CAP peak corresponding to unmyelinated and demyelinated axons was detected at 4, 7, and 14 dpi (conduction velocities: 0.25 ± 0.02 m.s^−1^ for 4 dpi (*n* = 6) and 0.29 ± 0.04 m.s^−1^ for 7 and 14 dpi (*n* = 5); Figure 7E). CAPs had variable amplitudes and were not significantly modified at any time point (Figure 7F). Altogether, our data demonstrate major changes in glutamatergic synaptic connectivity of OPCs in demyelinated lesions, independent on the ability of axons to conduct action potentials.

## Discussion

The major finding of this study is that important alterations of the synaptic connectivity between neurons and OPCs occur following demyelination, which could have a physiopathological relevance in demyelinating diseases. Whereas, virtually all recorded OPCs display Na^+^ currents and no changes in voltage-independent K^+^ conductances, newly-generated OPCs do not receive synaptic inputs during their active proliferation after demyelination, but gain synapses during the remyelination process. Importantly, we also demonstrated the presence of axonal-OPC contacts in active MS lesions.

We showed that transgenic lines constitute a powerful tool for a reliable identification of OPCs in acute slices, but that they have to be used with pertinence since the expression of fluorescent reporters was not fully characterized in the adult brain (see Supplementary Figures 1A,D; see also Clarke et al., 2012). Herein, our data implies that activated microglia expressed NG2 under acute demyelinating conditions (see also Bu et al., 2001; Zhu et al., 2012) that compromised the discrimination of OPCs in brain slices of lesioned NG2-DsRed mice. The expression of the NG2 proteoglycan in microglia has been underestimated in previous studies and our result clearly indicate that this marker should be used with caution for the identification of OPCs during patch-clamp recordings in acute demyelinating lesions. Moreover, our analysis revealed that GFP was not exclusively expressed in OPCs, but was also detected in mature oligodendrocytes in the PDGFRα-GFP transgenic mouse brain. Therefore, the combination of different transgenic mouse lines, immunohistological and electrophysiological analysis are required to overcome pitfalls and allowed for a reliable identification of OPCs in normal and lesioned *corpus callosum* of adult animals.

Interestingly, capacitance, Na^+^ currents and voltage-independent K^+^ conductances of OPCs in the normal and lesioned adult *corpus callosum* resemble those described previously in white matter OPCs recorded in juvenile animals (Chittajallu et al., 2004; De Biase et al., 2010). On the contrary, gray matter OPCs undergo important developmental modifications of their I-V curves, conferred by the postnatal upregulation of Kir4.1 channels (Maldonado et al., 2013). Differences between OPCs of gray and white matter regions are not restricted to electrophysiological and anatomical properties. It was recently demonstrated that the environmental “niche” from where OPCs belong determines their differentiation properties (Vigano et al., 2013). OPCs derived from white matter differentiate into myelinating oligodendrocytes, independently if they are transplanted to white or gray matter regions, whereas OPCs derived from gray matter did not. The location of OPCs seems therefore to confer intrinsic differences. These distinct differentiation properties of OPCs from gray and white matter strengthens the idea of the existence of heterogeneous OPC populations and suggests that the role play by these cells diverge according to brain regions. While OPCs are probably not progenitors only in the adult gray matter (Maldonado et al., 2013), they renew the population of oligodendrocytes throughout life in healthy and pathological conditions in white matter (Franklin and Ffrench-Constant, 2008; Young et al., 2013).

Our electrophysiological and immunohistochemical results revealed important alterations of OPC glutamatergic connectivity in demyelinated lesions, which could reflect physiological changes in reactivated OPCs induced by demyelination. The existence of two OPC populations, defined by the presence or absence of Na^+^ channels and of axonal synaptic inputs, has been previous suggested in the cerebellar white matter (Karadottir et al., 2008). Yet, the existence of these two distinct subpopulations of OPCs was contradicted in more recent reports arguing that this heterogeneity was due to the recording of pre-oligodendrocytes that rapidly lose both their functional Na^+^ channels and synapses (De Biase et al., 2010; Etxeberria et al., 2010; Kukley et al., 2010; see also Maldonado et al., 2011). Nevertheless, though most studies agree with the idea that all OPCs express Na^+^ conductances in physiological conditions, no reports have addressed this question in demyelinating lesions. Since OPCs do not necessarily have the same electrophysiological properties in young and adult mice (Zhou et al., 2006; Vélez-Fort et al., 2010; Balia et al., 2013; Maldonado et al., 2013), this electrophysiological criterion needed to be tested after white matter demyelination. We concluded that virtually all OPCs in the adult *corpus callosum* express Na^+^ channels either in control or in demyelinating lesions and thus that this property can be considered as a hallmark. We also confirmed that all OPCs in normal white matter are synaptically contacted by axons as previously reported (De Biase et al., 2010). However, axon-OPC synaptic connectivity following demyelination undergoes a pronounced down-regulation during the reactivation and active proliferation of endogenous OPCs, which results in a transient decrease in the number of connected OPCs in LPC-induced lesions.

Sensory experience has recently been shown to control thalamic innervation of OPCs during early postnatal development of the barrel cortex (Mangin et al., 2012). In line with the effect of sensory deprivation on the reduction of thalamocortical inputs and the increase in cortical OPC proliferation (Mangin et al., 2012), our data showed that reactivated OPCs are poorly connected at 4 dpi during their active proliferation following demyelination. Therefore, synaptic loss is concomitant with OPC proliferation in different conditions, indicating that OPC innervation is a dynamic feature that changes according to neuronal activity and cell environment in different brain regions. A role for glutamatergic synaptic activity in preventing proliferation has been shown in organotypic slice cultures (Yuan et al., 1998). However, dividing OPCs appear to retain afferent synapses and share them with daughter cells during postnatal development (Kukley et al., 2008; Ge et al., 2009). While mitotic OPCs have a reduced synaptic activity compared to non-mitotic cells (Kukley et al., 2008), this discrepancy seems contradictory. We also showed that reactivated OPCs in LPC-induced lesions receive sparse AMPA receptor-mediated synapses, but gain synaptic inputs after their active proliferation following demyelination. Therefore, even if glutamatergic inputs to OPCs do not function exclusively to inhibiting proliferation, they seem likely to influence this process *via* glutamate release. One possibility of a causal link between synaptic activity and proliferation is that AMPA receptor activation by synaptic glutamate release locally increases intracellular calcium concentrations in thin OPC processes (see Maldonado and Angulo, 2014 for discussion). In turn, glutamate-dependent calcium elevations might activate a signaling pathway controlling gene expression, as observed in cell culture (Pende et al., 1994), and inhibiting developmental OPC progression. In line with this, we speculate that the decrease of glutamatergic axon-OPC synapses early after demyelination impairs glutamate-dependent calcium signals, facilitating OPC proliferation and having paradoxically a beneficial effect in demyelinating conditions.

### Conflict of interest statement

The authors declare that the research was conducted in the absence of any commercial or financial relationships that could be construed as a potential conflict of interest.

## Supplementary material

Supplementary Figure 1 **Characterization and electrophysiological properties of GFP+ cells in the PDGFRα-GFP transgenic line in control conditions. (A)** Sagittal brain section through the *corpus callosum* of 2 month-old control NG2-DsRed mouse. At the adult stage, the expression of DsRed is only detected in pericytes around blood vessels (arrows). **(B,C)** NG2+/GFP+ OPCs (**B**, arrows) and CC1+/GFP+ differentiated oligodendrocytes (**C**, arrows) in PDGFRα-GFP mouse strain. **(D)** Histogram showing the percentage of NG2+ and CC1+ cells in control *corpus callosum* in the PDGFRα-GFP strains (*N* = 4 mice). ^**^*p* < 0.01 respect to Olig2 expression. **(E,F)** Currents induced by voltage steps from +40 to −120 mV in an OPC **(E)** and an oligodendrocyte **(F)** held at −90 mV in a PDGFRαR-GFP mouse. Note the absence of INa^+^ in the oligodendrocyte (inset) and the difference in the scale bars. **(G)** Histogram of the proportion of OPCs (O) and oligodendrocyte (OL) identified by their electrophysiological profiles and recorded in PDGFRα-GFP mouse strain. It is noteworthy that patch-clamp recordings in the demyelinated *corpus callosum* revealed current profiles typical of mature oligodendrocytes in 17 of 18 GFP+ recorded cells in this mouse line, precluding the identification of OPCs in lesions.

Supplementary Figure 2 **Characterization of NG2+ cells in MS lesions. (A,B)** Immunohistochemistry of Olig1 (red) and NG2 (green, **A**) or Iba1 (green, **B**) labeling in an active zone of a MS lesion. Nuclei were stained with Dapi (blue).
