# Supplementary figures and images for: Alteration of synaptic connectivity of oligodendrocyte precursor cells following demyelination
Source: Front Cell Neurosci. 2015 Mar 17;9:77. doi: 10.3389/fncel.2015.00077 (PMC4362325; doi:10.3389/fncel.2015.00077)

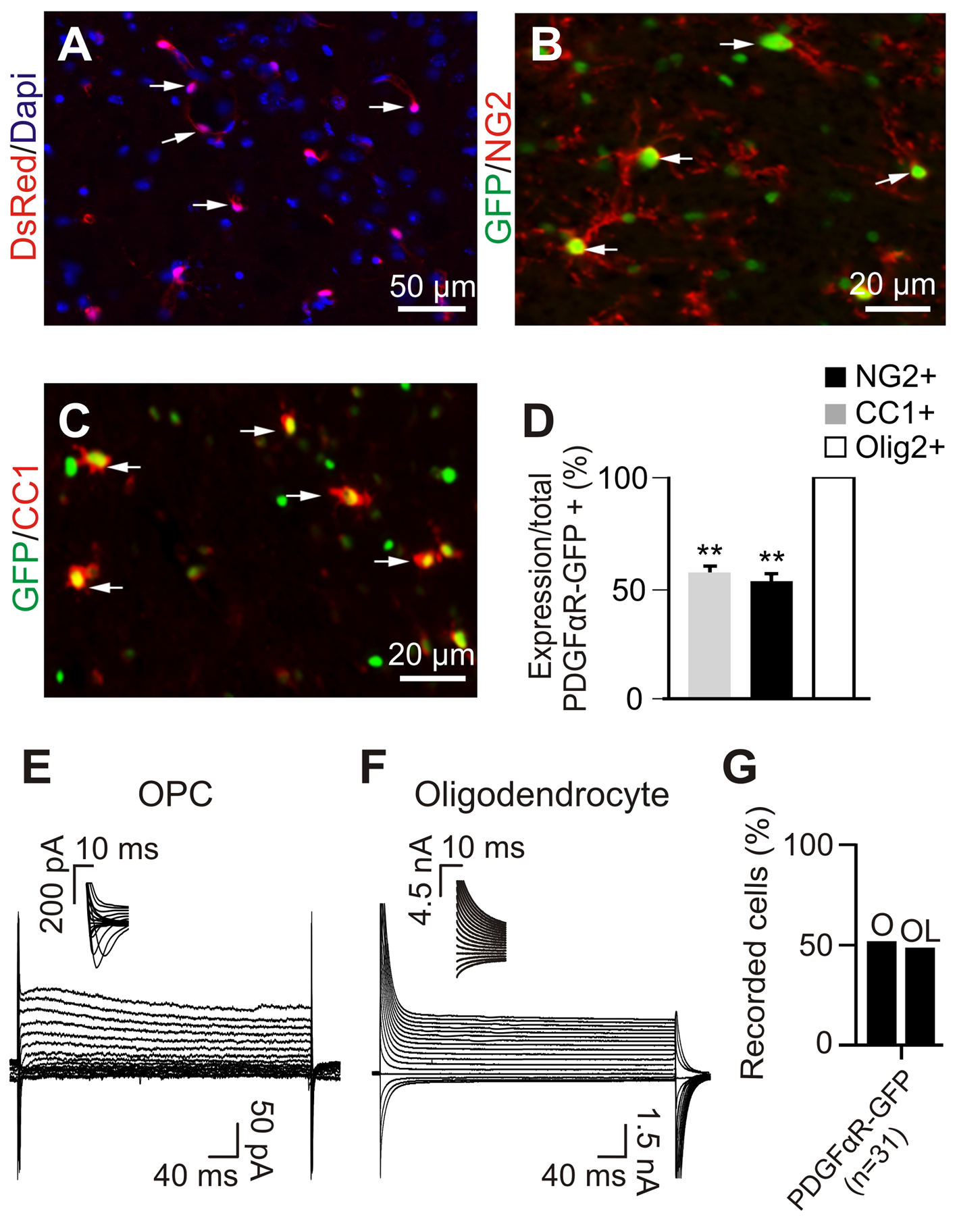

Supplement: Supplementary Figure 1 — Characterization and electrophysiological properties of GFP+ cells in the PDGFRα-GFP transgenic line in control conditions. (A) Sagittal brain section through the corpus callosum of 2 month-old control NG2-DsRed mouse. At the adult stage, the expression of DsRed is only detected in pericytes around blood vessels (arrows). (B,C) NG2+/GFP+ OPCs (B, arrows) and CC1+/GFP+ differentiated oligodendrocytes (C, arrows) in PDGFRα-GFP mouse strain. (D) Histogram showing the percentage of NG2+ and CC1+ cells in control corpus callosum in the PDGFRα-GFP strains (N = 4 mice). **p < 0.01 respect to Olig2 expression. (E,F) Currents induced by voltage steps from +40 to −120 mV in an OPC (E) and an oligodendrocyte (F) held at −90 mV in a PDGFRαR-GFP mouse. Note the absence of INa+ in the oligodendrocyte (inset) and the difference in the scale bars. (G) Histogram of the proportion of OPCs (O) and oligodendrocyte (OL) identified by their electrophysiological profiles and recorded in PDGFRα-GFP mouse strain. It is noteworthy that patch-clamp recordings in the demyelinated corpus callosum revealed current profiles typical of mature oligodendrocytes in 17 of 18 GFP+ recorded cells in this mouse line, precluding the identification of OPCs in lesions. [file Image1.TIF]

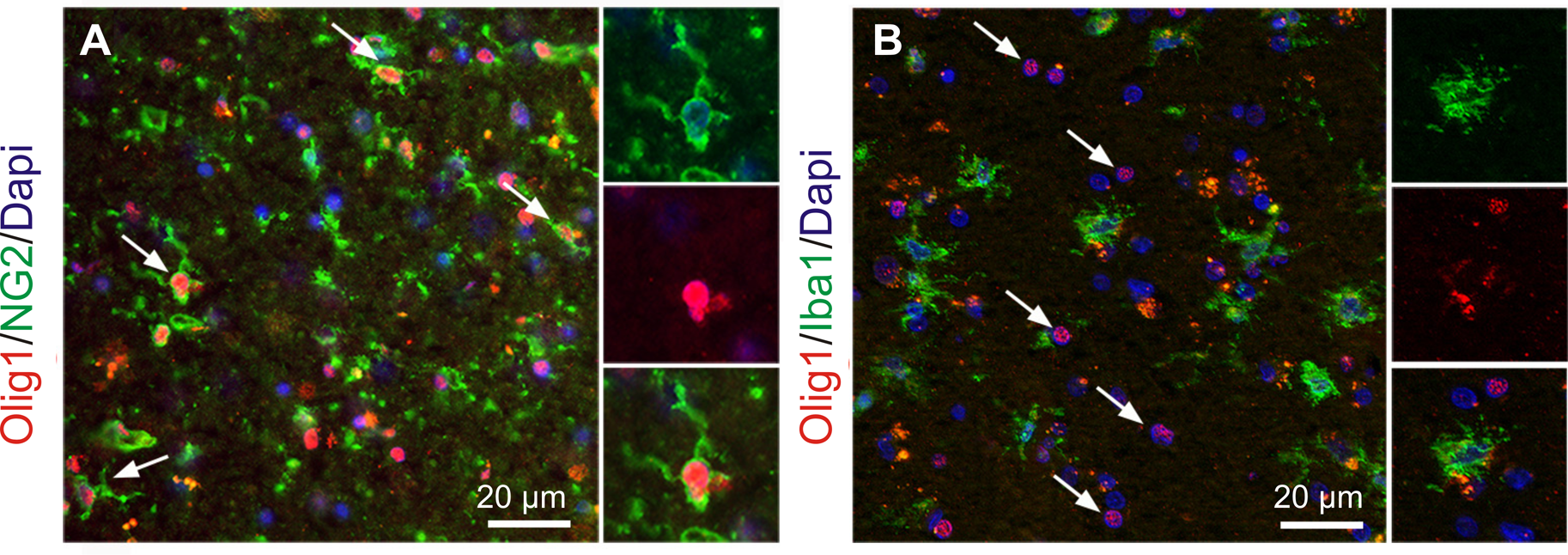

Supplement: Supplementary Figure 2 — Characterization of NG2+ cells in MS lesions. (A,B) Immunohistochemistry of Olig1 (red) and NG2 (green, A) or Iba1 (green, B) labeling in an active zone of a MS lesion. Nuclei were stained with Dapi (blue). [file Image2.TIF]
